# The Impact of PNPLA3 rs738409 SNP on Liver Fibrosis Progression, Portal Hypertension and Hepatic Steatosis in HIV/HCV Coinfection

**DOI:** 10.1371/journal.pone.0143429

**Published:** 2015-11-23

**Authors:** Bernhard Scheiner, Mattias Mandorfer, Philipp Schwabl, Berit Anna Payer, Theresa Bucsics, Simona Bota, Maximilian C. Aichelburg, Katharina Grabmeier-Pfistershammer, Albert Stättermayer, Peter Ferenci, Michael Trauner, Markus Peck-Radosavljevic, Thomas Reiberger

**Affiliations:** 1 Division of Gastroenterology and Hepatology, Department of Internal Medicine III, Medical University of Vienna, Vienna, Austria; 2 Vienna HIV & Liver Study Group, Medical University of Vienna, Vienna, Austria; 3 Division of Immunology, Allergy and Infectious Diseases, Department of Dermatology, Medical University of Vienna, Vienna, Austria; University of Sydney, AUSTRALIA

## Abstract

**Background:**

Faster fibrosis progression and hepatic steatosis are hallmarks of HIV/HCV coinfection. A single nucleotide polymorphism (SNP) of the PNPLA3-gene is associated with development of non-alcoholic steatohepatitis and a worse outcome in alcoholic liver disease. However, the role of PNPLA3 rs738409 SNP on liver fibrosis and steatosis, portal hypertension, and virological response in HIV/HCV coinfection remains unclear.

**Methods:**

In this cross-sectional study PNPLA3 (rs738409) and IL28B (rs12979860) SNPs were determined in 177 HIV/HCV coinfected patients. Liver fibrosis and steatosis—staged by liver biopsy and transient elastography using the Controlled Attenuation Parameter (CAP)–and portal hypertension (hepatic venous pressure gradient, HVPG) were compared across PNPLA3 genotypes.

**Results:**

75 (42.4%) patients tested positive for a PNPLA3 minor/major risk allele (G/C:66; G/G:9) showed comparable fibrosis stages (median F2 vs. F2; p = 0.292) and similar amounts of hepatic steatosis (CAP: 203.5±41.9 vs. 215.5±59.7dB/m; p = 0.563) as compared to patients without a PNPLA3 risk allele. Advanced liver fibrosis was neither associated with PNPLA3 (p = 0.253) nor IL28B-genotype (p = 0.628), but with HCV-GT3 (p = 0.003), higher BMI (p = 0.008) and higher age (p = 0.007). Fibrosis progression rate (0.27±0.41 vs. 0.20±0.26 units/year; p = 0.984) and HVPG (3.9±2.6 vs. 4.4±3.0 mmHg; p = 0.472) were similar in patients with and without PNPLA3 risk alleles. SVR rates to PEGIFN/RBV therapy were similar across PNPLA3 genotypes.

**Conclusions:**

The presence of a PNPLA3 risk allele had no independent impact on liver disease or virological response rates to PEGIFN/RBV therapy in our cohort of HIV/HCV coinfected patients.

## Introduction

Worldwide, more than 4 million HIV-positive individuals are coinfected with hepatitis C virus (HCV), leading to significantly increased morbidity and mortality [1,2]. When compared to patients with HCV monoinfection [3], patients with HIV/HCV coinfection show accelerated fibrosis progression [4,5] and a higher risk of developing life-threatening complications such as end-stage-liver-disease (ESLD) and hepatocellular carcinoma (HCC). Highly effective direct-acting antiviral (DAA)-based therapies against HCV infection [6,7] are currently often restricted to high risk patients due to limited resources in many health care settings [8]. Thus, stratifying patients by their individual risk of developing advanced liver disease represents an important clinical challenge [9]. Several risk factors for accelerated fibrosis progression rates—such as IL28B C/C-genotype [10,11]—also referred to as IFNL4, low CD4+ cell counts [5], uncontrolled HIV-infection [12], HCV-genotype 3 [13] and low 25(OH)D levels [14]–have previously been identified.

Even though it has been studied intensively in different settings within the last 6 years [15], the influence of a genetic polymorphism in the patatin-like phospholipase domain-containing 3 (PNPLA3)-gene (rs738409) on liver disease progression in HIV/HCV coinfected patients remains unclear. In contrast to the wild-type, in vitro experiments showed that the mutated PNPLA3-protein lost its triglyceride lipase activity leading to increased triglyceride accumulation in Huh-7 cells and thereby leading to a two-fold increase in hepatic fat [16]. Furthermore PNPLA3 is thought to possess acylglycerol transacetylase activity [17]. Even though the main mechanism of PNPLA3 in vivo is not completely understood, Romeo et al. described its influence on the development of hepatic steatosis and hepatic necroinflammation, thereby increasing the susceptibility for non-alcoholic fatty liver disease (NAFLD) [18]. Recent publications showed a significantly higher risk for hepatic steatosis, more pronounced necroinflammation and an accelerated fibrosis progression rate in HCV monoinfected patients harbouring the PNPLA3 risk allele G [19–21]. Another study confirmed these findings in HIV monoinfected patients showing a strong correlation between PNPLA3 non-C/C genotype and hepatic steatosis [22]. Moreover, Trépo et al. reported a 2.5 times faster fibrosis progression in HCV monoinfected patients harbouring the major risk genotype (G/G) [23]. The prevalence of the PNPLA3 minor risk genotype (G/C) is reported to be rather high with 36.8–49.2%, while the prevalence of the PNPLA3 major risk genotype (GG) is reported to be 2.2–22.2% in patients with HCV monoinfection [19,20,23]. Thus, the PNPLA3 polymorphism seems to play a relevant role in fibrosis progression of patients with chronic hepatitis C (CHC) [20,21]. To our best knowledge only one previous study from our group [24] examined the influence of PNPLA3 on liver fibrosis progression in HIV/HCV coinfection, but the power of this study was limited by the low number of patients with PNPLA3 G/G risk alleles.

We aimed to assess the impact of PNPLA3 SNP (rs738409) on (i) fibrosis progression rate and development of advanced fibrosis, (ii) liver steatosis, (iii) portal hypertension, and (iv) virological response to PEGIFN/RBV therapy in a large cohort of HIV/HCV coinfected patients.

## Patients and Methods

### Study population (Fig 1)

**Fig 1:**
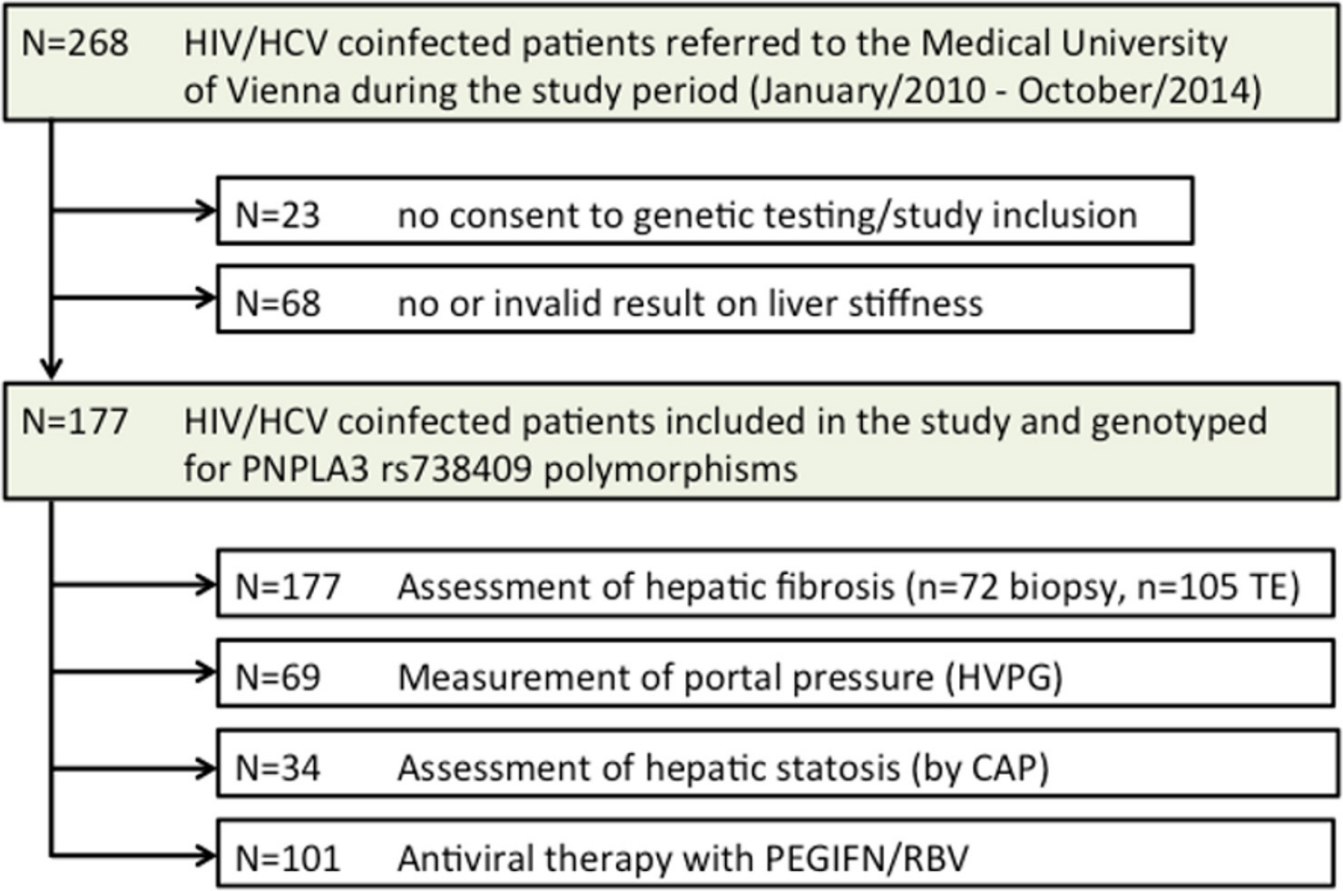
Patient flowchart showing the number of HIV/HCV coinfected patients referred to the Medical University of Vienna, the number of patients included / excluded from the study and the number of patients undergoing assessment of hepatic fibrosis and steatosis as well as portal pressure and the number of patients treated with PEGIFN/RBV; Abbreviations: TE (transient elastography), HVPG (hepatic venous pressure gradient), CAP^™^ (Controlled Attenuation Parameter).

HIV/HCV coinfected patients referred to the Medical University of Vienna between January 2010 and October 2014 were enrolled in this retrospective study if the following criteria were fulfilled (i) proven chronic HCV- (HCV-RNA detectable) and HIV-infection (anti-HIV1/2 positive), (ii) available liver biopsy or valid measurement of liver stiffness by transient elastography, (iii) being HCV treatment naïve at the time of liver biopsy or transient elastography, and (iv) available genetic testing for PNPLA3 (rs738409) SNP. Patients with other concomitant liver diseases were excluded from this study. HVPG-measurements were performed in 69 patients, CAP^™^-measurement for evaluation of hepatic steatosis in 34 patients.

### Genotyping, blood tests and definitions

PNPLA3 (rs738409) and IL28B (rs12979860) genotyping was performed as previously described [25] using the StepOne-Plus Real Time PCR System and a Custom TaqMan SNP Genotyping Assay (Applied Biosystems, Carlsbad, CA, USA) [21]. Alcohol abuse was defined as self-reported actual or former daily alcohol intake of >50g for more than 2 consecutive years. HCV genotype and serum HCV-RNA levels were evaluated using the VERSANT HCV Genotype 2.0 Assay (LiPA) (Siemens, Vienna, Austria) and the COBAS TaqMan HCV Test (Roche, Vienna, Austria). High HCV load was defined by serum HCV-RNA levels >6x10^5^ IU/mL similar to Neukam et al. showing that HIV/HCV-coinfected patients presenting with high HCV load have a lower probability of treatment response to PEGIFN/RBV [26]. High levels of aminotransferases (AST/ALT) were defined as >2x gender specific upper limit of normal (ULN).

### Staging of fibrosis and steatosis and calculation of fibrosis progression rate

Fibrosis was determined either by liver biopsy (n = 72) or transient elastography (n = 105) (FibroScan, Echosens, France). Advanced fibrosis was defined as METAVIR F3/F4 [27] or liver stiffness >9.5kP [28,29]. Fibrosis progression rate (FPR) was calculated as METAVIR F-units divided by years since the estimated year of infection. Steatosis was evaluated using the Controlled Attenuation Parameter (CAP^™^) module of the FibroScan^®^ device [30]. According to M. Sasso et al. a CAP^™^-value ≥222 dB/m identifies patients with significant steatosis (≥S1 according to METAVIR) [30].

### Measurement of portal pressure by the hepatic venous pressure gradient (HVPG)

Physiological HVPG is usually between 2–5mmHg in healthy individuals—therefore elevated portal pressure is defined as HVPG ≥6mmHg [31]. Reports showed that a minimal portal pressure of 10–12mmHg is required for the development of varices—therefore clinically significant portal hypertension (CSPH) is defined as HVPG ≥10mmHg [32].

### Treatment of Chronic Hepatitis C

Virological response rates were available in 101 patients who underwent antiviral therapy with PEGIFN/RBV.

### Statistical analysis

Statistical analyses were performed using IBM SPSS Statistics 22 (SPSS Inc., Vienna, Austria). Continuous variables are reported as mean±standard deviation or median (interquartile range (IQR)). Categorical variables are shown as numbers (proportions) of patients with the respective characteristics. Pearson’s chi-squared test or Fisher’s exact test were used for group comparisons of categorical variables, while continuous variables were analysed by Mann-Whitney-U-test or Student’s t-test, when applicable. Binary logistic regression analysis was used for determination of factors independently associated with advanced fibrosis. A p-value ≤0.05 was considered statistically significant. A power analysis was performed based on the data from a previous study by Trepo et al. [23] assessing the impact of PNPLA3 on fibrosis progression in HCV monoinfected patients indicating advanced fibrosis (METAVIR F3 or F4) in 60.5% of G/G patients and 38.7% of C/C and C/G patients (dominant model). Defining an alpha error (0.05) and a power (1-ß) of 0.80, the power analysis computed a required sample size of 174 patients. As a recent publication [33] showed no influence of PNPLA3 SNP on liver disease progression in HCV-GT3 patients, we repeated all analyses for the cohort without including HCV-GT3 patients (n = 38).

### Ethics

This study was conducted with approval of the ethics committee of the Medical University of Vienna (EK 1369/2012) and written informed consent was obtained from all patients.

## Results

### Patient characteristics (Table 1)

**Table 1:** Patient characteristics according to PNPLA3 genotypes.

		PNPLA3 SNP (rs738409)		
Patient characteristics	All patients, n = 177	PNPLA3 C/C, n = 102	PNPLA3 G/G or G/C, n = 75	p-value
**Sex (M/F, % male)**	134/43 (75.7%)	81/21 (79.4%)	53/22 (70.7%)	0.180
**Age (years)**	38.60±9.92	38.36±9.59	38.92±10.42	0.638
BMI (kg*m ^**-2**^ **)**	23.13±4.25	23.38±4.73	22.81±3.53	0.846
**HCV-Transmission**				0.978
***IVDU***	129 (72.9%)	76 (74.5%)	53 (70.7%)	
***MSM***	34 (19.2%)	18 (17.6%)	16 (21.3%)	
***Heterosexual***	7 (4.0%)	4 (2.0%)	3 (6.7%)	
***Transfusion/Blood products***	5 (2.8%)	3 (2.9%)	2 (2.7%)	
***Unknown***	2 (1.1%)	1 (1.0%)	1 (1.3%)	
**Years between infection and fibrosis evaluation**	11.5±10.01	11.29±9.21	11.80±11.08	0.882
**HCV Genotype**				0.106
***GT 1***	113 (63.8%)	59 (57.8%)	54 (72.0%)	
***GT 2***	3 (1.7%)	2 (2.0%)	1 (1.3%)	
***GT 3***	38 (21.5%)	26 (25.5%)	12 (16.0%)	
***GT 4***	21 (11.9%)	15 (14.7%)	6 (8.0%)	
***GT 6***	2 (1.1%)	0 (0%)	2 (2.7%)	
High HCV-RNA (>6*10 ^**5**^ **IU/mL)**	118 (66.7%)	65 (63.7%)	53 (70.7%)	0.333
**Current cART**	147 (83.1%)	83 (81.4%)	64 (85.3%)	0.677
**HIV-RNA level (log10 copies/mL)**	1.91±1.06	1.91±1.11	1.89±0.98	0.441
**Alcohol abuse**	42 (23.7%)	26 (25.5%)	16 (21.3%)	0.452
**CD4 count (cells/μL)**	528.1±263.0	538.4±269.3	514.5±255.5	0.700
**CD4 percentage**	28.89±10.12%	28.46±10.42%	29.44±9.76%	0.364
**CD4 nadir (cells/μL)**	274±200	285±201	258±200	0.331
**ALT (IU/mL)**	88.7±82.2	74.1±60.0	109.0±103.5	**0.021**
**High ALT (>2xULN)**	59 (33.3%)	26 (25.5%)	33 (44.0%)	**0.010**
**AST (IU/mL)**	66.5±43.7	60.5±41.3	74.8±45.8	**0.033**
**High AST (>2xULN)**	43 (24.3%)	18 (17.7%)	25 (33.3%)	**0.016**
**y-GT (IU/mL)**	127.4±117.1	113.5±106.0	146.5±129.3	**0.059**
**IL28B-genotype**				0.248
***C/C***	57 (32.2%)	30 (29.4%)	27 (36.0%)	
***T/C***	101 (57.1%)	63 (61.8%)	38 (50.7%)	
***T/T***	18 (10.2%)	8 (7.8%)	10 (13.3%)	
**IL28B non-C/C**	119 (67.2%)	71 (69.6%)	48 (64.0%)	0.468
**Fibrosis according to METAVIR**				0.292
***F0***	31 (17.5%)	18 (17.6%)	13 (17.3%)	
***F1***	45 (25.4%)	28 (27.5%)	17 (22.7%)	
***F2***	58 (32.8%)	28 (27.5%)	30 (40.0%)	
***F3***	20 (11.3%)	15 (14.7%)	5 (6.7%)	
***F4***	23 (13.0%)	13 (12.7%)	10 (13.3%)	
**Liver Stiffness (kPa; n = 105)**	6.5 (3.7)	6.4 (3.9)	6.8 (3.3)	0.334
**Fibrosis Progression Rate (METAVIR F-units/year)**	0.23±0.33	0.20±0.26	0.27±0.41	0.984
**CAP (dB/m; n = 34)**	209.5±51.1	215.5±59.7	203.5±41.9	0.563
**HVPG (mmHg; n = 69)**	4.2±2.8	4.4±3.0	3.9±2.6	0.472
**Portal Hypertension (HVPG≥6mmHg)**	13 (18.8%)	10 (9.8%)	3 (4.0%)	0.124

Patient characteristics (demographic data, data on HIV/HCV coinfection, IL28B-genotype, data on liver fibrosis, hepatic steatosis, liver stiffness, fibrosis progression rate and hepatic venous pressure gradient) according to PNPLA3 (patatin-like phospholipase domain-containing protein 3)-risk allele; continuous variables shown as mean ± SD or median (IQR); Abbreviations: SNP (single nucleotide polymorphism), IVDU (intravenous drug-use), MSM (men having sex with men), CAP^™^ (Controlled Attenuation Parameter), HVPG (hepatic venous pressure gradient).

One hundred seventy-seven HIV/HCV coinfected patients were included. Out of those 70 (39.5%) patients were also included in the previous study of our group [24]. The majority of patients was male (75.7%) with a mean age of 38.6±9.9 years and BMI of 23.1±4.3 kg*m^-2^. Forty-two patients (23.7%) had a history of or current alcohol abuse. The most common route of infection was intravenous drug-use (IVDU, 72.9%) with a mean duration of infection of 11.5±10.0 years. Most patients (66.7%) had high levels of HCV-RNA (>6*10^5^ IU/mL) and were on cART (83.1%) and thus, showed low HIV viremia (1.91±1.1 log10 copies/mL). Immune status was well preserved as reflected by a mean CD4+ T lymphocyte (CD4+) cell count of 528±263 cells/μL.

In regard to PNPLA3, nine patients (5.1%) were tested positive for the major PNPLA3 risk genotype (G/G), 66 (37.3%) for the minor risk genotype (G/C) and 102 (57.6%) had no risk allele (C/C). The distribution of IL28B genotype was: 18 (10.2%), 101 (57.1%) and 57 (32.2%) for T/T, T/C and C/C, respectively.

### Liver disease parameters (Table 1, Fig 2)

**Fig 2:**
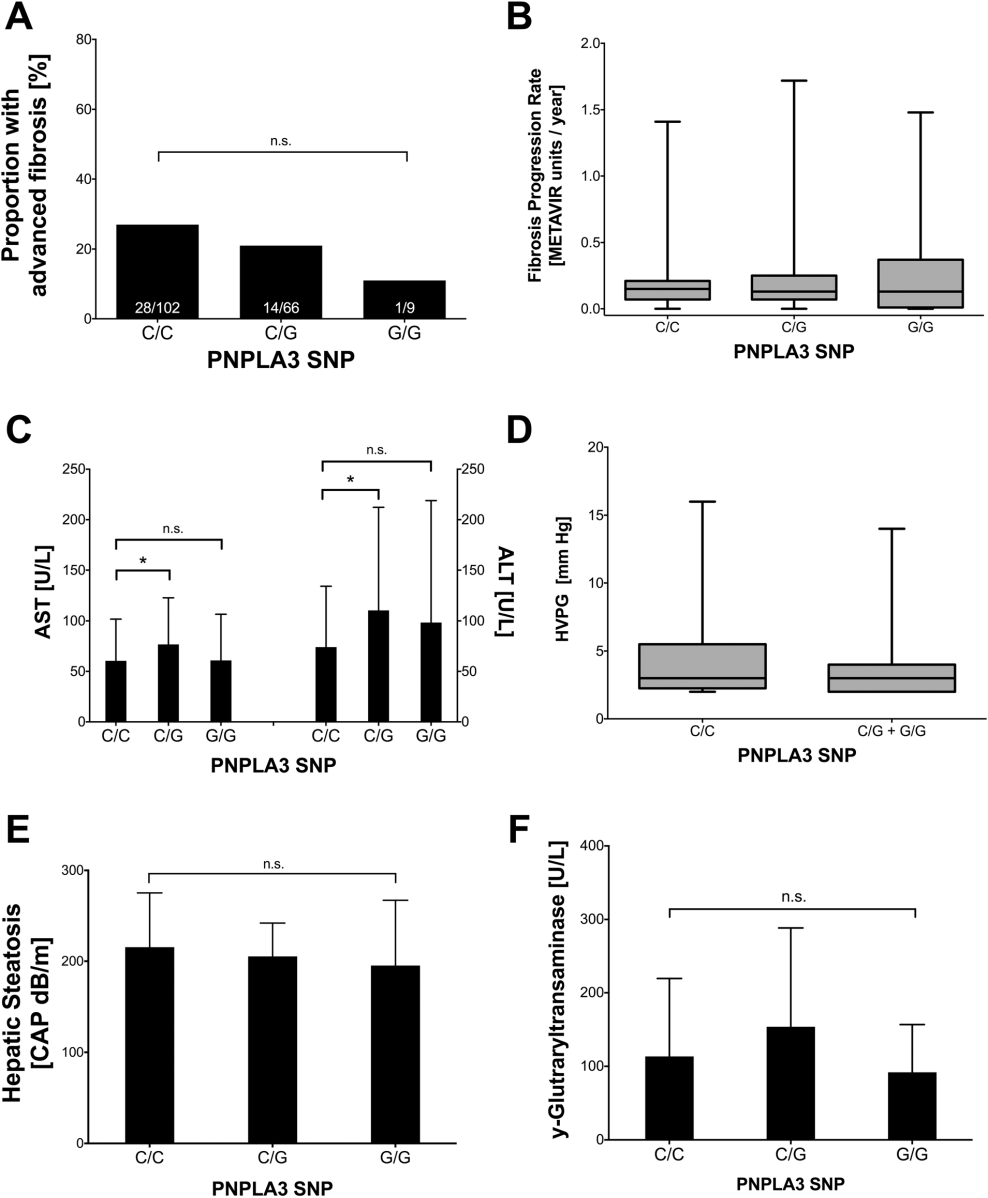
**A:** Proportion of patients with METAVIR F3/F4 according to PNPLA3-SNP. **B:** Fibrosis progression rate according to PNPLA3-SNP. **C:** Levels of aminotransferases according to PNPLA3 genotype. **D:** Portal pressure (HVPG) according to PNPLA3 genotype. **E:** Hepatic steatosis (assessed by CAP) according to PNPLA3 genotype. **F:** Levels of y-glutaryl transaminases according to PNPLA3 genotype. Abbreviations: PNPLA3 (patatin-like phospholipase domain-containing protein 3), SNP (single nucleotide polymorphism), HVPG (hepatic venous pressure gradient), CAP^™^ (Controlled Attenuation Parameter).

Thirty-one (17.5%) patients had METAVIR F0 fibrosis, 45 (25.4%) F1, 58 (32.8%) F2, 20 (11.3%) showed bridging fibrosis (F3), and 23 (13.0%) had established cirrhosis (F4). The mean fibrosis progression rate (FPR) was 0.23±0.33 METAVIR F-units/year. Mean AST and ALT were 66.5±43.7 IU/mL and 88.7±82.8 IU/mL, respectively. The majority of patients showed elevated levels of AST (60.0%) and ALT (65.5%).

34 patients (19.2%) underwent non-invasive steatosis assessment showing CAP results of 209.5±51.1dB/m. HVPG was measured in 69 (39.0%) patients who had a mean HVPG value of 4.2±2.8mmHg. Elevated portal pressure (HVPG≥6mmHg) was observed in 13 patients (18.8%) and clinically significant portal hypertension (HVPG ≥10mmHg) in 4 patients (5.8%) [34].

### Influence of PNPLA3 risk alleles on liver disease (Table 1, Fig 2)

Almost half of the patients (n = 75, 42.4%) were tested positive for a PNPLA3 major or minor risk allele (G/G or G/C). Most importantly, the prevalence of established risk factors for advanced fibrosis or accelerated fibrosis progression (such as prior alcohol abuse, HCV-GT3, low CD4+ nadir, etc.) was comparable between patients with and without PNPLA3 risk alleles. The (estimated) duration of HCV infection was similar in patients with PNPLA3 C/C, when compared to patients with PNPLA3 C/G or G/G with 11.3±9.2 and 11.8±11.1 years, respectively (p = 0.88). Patients with a PNPLA3 risk allele showed significantly higher ALT-levels (109.0±103.5 vs. 74.1±60.0 IU/mL; p = 0.02) and AST-levels (74.8±45.8 vs. 60.5±41.3 IU/mL; p = 0.03). Moreover, there was a trend toward higher y-GT-levels (146.5±129.3 vs. 113.5±106.0 IU/mL; p = 0.06) when compared to patients without a PNPLA3 risk allele. There was no difference in METAVIR fibrosis stage (both median F2 (range 1–4; p = 0.11)), nor mean liver stiffness (median 6.8 (3.3) vs. 6.4 (3.9)kPa; p = 0.33) between patients with and without a PNPLA3 risk allele.

### Comparison of PNPLA3 risk allele prevalence between patients with or without advanced fibrosis

One fourth of patients (n = 43, 24.3%) had advanced fibrosis (F3/F4). Not surprisingly, patients with advanced fibrosis showed a significantly longer duration of infection (16.2±9.6 vs. 10.0±9.7 years; p<0.01), a significantly lower CD4+ cell count (465±271 vs. 549±258cells/μL; p = 0.02), a significantly lower CD4-nadir (207±166 vs. 296±206 cells/μL; p = 0.01), and lower HDL-Cholesterol (40±17 vs. 49±18mg/dL; p<0.01). HCV-GT 3 was overrepresented in the group of patients with advanced fibrosis (42% vs. 15%; p<0.01). Patients with advanced fibrosis were more likely to be subsequently treated with PEGIFN/RBV than patients without advanced fibrosis (72% vs. 52%; p = 0.02). However, the distribution of PNPLA3 non-C/C (44.8% vs. 34.9%; p = 0.25) and IL28B non-C/C (68.7% vs. 62.8%; p = 0.63) SNPs was comparable between both groups.

### Multivariable analysis of factors independently associated with advanced fibrosis (METAVIR F3/F4) (Table 2)

**Table 2:** Factors independently associated with advanced fibrosis (F3/F4).

Patient characteristics	F0/F1/F2 n = 134	F3/F4 n = 43	UVA p-value	Odds Ratio	MVA p-value
**Sex [M/F, % male]**	103/31 (76.9%)	31/12 (72.1%)	0.525	-	-
**Age (years)**	37.04±9.73	43.46±9.00	<0.01	**1.87 (1.19–2.94)**	**0.007**
BMI (kg*m ^**-2**^ **)**	22.81±4.06	24.14±4.68	0.127	**1.14 (1.04–1.26)**	**0.008**
**Duration of infection**	9.99±9.73	16.19±9.50	<0.01	1.16 (0.66–2.05)	0.600
**HCV-GT3 [n, %]**	20 (14.9%)	18 (41.9%)	<0.01	**4.18 (1.62–10.80)**	**0.003**
**HCV-RNA (log10 IU/mL)**	5.99±1.21	6.29±0.88	0.261	1.19 (0.80–1.78)	0.386
High HCV-RNA (>6*10 ^**5**^ **IU/mL)**	85 (63.4%)	33 (76.7%)	0.107	-	-
**Current cART**	107 (79.9%)	40 (93.0%)	0.063	-	-
**Time on cART**	2.92 (5.22)	4.2 (7.56)	0.470	-	-
**CD4 nadir (cells/μL)**	296±206	207±166	0.011	**0.80 (0.63–1.01)**	**0.058**
**ALT (IU/mL)**	89.2±87.7	87.2±65.8	0.572	-	-
**AST (IU/mL)**	62.8±43.2	78.0±43.9	0.016	1.02 (0.92–1.14)	0.679
**Alcohol Abuse [n, %]**	27 (20.1%)	15 (34.9%)	0.058	1.41 (0.56–3.58)	0.466
**PNPLA3 G-allele [n, %]**	60 (44.8%)	15 (34.9%)	0.253	0.45 (0.28–1.76)	0.446
**IL28B non-C/C genotype [n, %]**	92 (68.7%)	27 (62.8%)	0.628	-	-

Factors associated with advanced fibrosis in HIV/HCV coinfection in an univariable as well as a multivariable analysis; factors in univariable analysis are shown as mean ± SD or number (percentage) of patients; on the right side all factors included in the multivariable analysis are shown, factors written in bold remained in the final model; Abbreviations: UVA (univariable analysis), MVA (multivariable analysis).

The binary logistic regression model (Model A) comprises variables, which were associated with advanced liver fibrosis in univariate analysis. In addition, we included well-established risk factors for liver disease progression in HCV-monoinfected patients as covariates. HCV GT 3 (OR: 4.18; 95% CI: 1.62–10.80; p = 0.003), higher age (OR: 1.87; 95% CI: 1.19–2.94; p = 0.007) and higher BMI (OR: 1.14; 95% CI: 1.04–1.26; p = 0.008) were independent risk factors for advanced liver fibrosis. Moreover, there was a trend toward an increased risk of advanced fibrosis in patients with low CD4 nadir (OR: 0.80; 95% CI: 0.63–1.01; p = 0.058). In contrast, advanced liver fibrosis was not associated with PNPLA3 G-allele (OR: 0.70; 95% CI: 0.28–1.76; p = 0.446), longer duration of infection (OR: 1.16; 95% CI: 0.66–2.05; p = 0.600), HCV-RNA levels (OR 1.19; 95% CI 0.80–1.78; p = 0.386), alcohol abuse (OR 1.41; 95% CI: 0.56–3.58; p = 0.466) and AST-levels (OR: 1.02; 95% CI: 0.92–1.14; p = 0.679). Thus, we did not observe an association between PNPLA3 and advanced liver fibrosis when adjusting for other relevant factors.

### Liver steatosis in patients with or without PNPLA3 risk allele (Table 1, Fig 2)

Liver steatosis—as assessed by CAP—was comparable between patients with PNPLA3 C/C and PNPLA3 non-C/C patients (215.5±59.7 vs. 203.5±41.9dB/m; p = 0.56).

### Subgroup analysis of patients with available HVPG-measurement

Sixty-nine (39.0%) patients underwent HVPG-measurement and almost half of those patients (n = 29, 42.0%) were tested positive for a PNPLA3 major or minor risk genotype. Harbouring a PNPLA3-risk allele in this subgroup of patients was associated with a non-significant trend towards a higher bilirubin levels (0.93±0.73 vs. 0.74±0.47 mg/dL;p = 0.07) as well as higher ALT levels (109.6±89.3 vs. 71.6±47.0; p = 0.09). The mean HVPG-value, however, was comparable between patients with and without a risk allele (3.9 ±2.6 vs. 4.4±3.0mmHg; p = 0.47).

Similarly to the results obtained in the entire cohort, there were no differences in presence of advanced fibrosis (24.1% vs. 42.5%; p = 0.11) and CAP-value (p = 0.27) between patients with and without PNPLA3 risk alleles.

### Influence of PNPLA3 on fibrosis progression, hepatic steatosis and HVPG after exclusion of HCV GT-3 patients

After excluding n = 38 HIV/HCV-GT3 patients for this subgroup analysis, the results were comparable to those obtained from the entire cohort with similar FPR (0.28±0.44 vs. 0.21±0.30, p = 0.838), similar amount of hepatic steatosis (CAP: 200.6±41.6 vs. 196.8±39.8, p = 0.851) and similar degree of portal hypertension (HVPG: 3.5±1.5 vs. 3.6±2.1, p = 0.752) across PNPLA3 genotypes.

### PNPLA3 genotype and virologic response to PEGIFN/RBV therapy (Table 3)

**Table 3:** Virological response to PEGIFN/RBV by PNPLA3-SNP and IL28B-SNP.

	PNPLA3			
	C/C	G/C	G/G	p-value
**IL28B C/C-patients (n = 28) [n]**	**16**	**8**	**4**	
*RVR*	4 (25.0%)	1 (12.5%)	2 (50.0%)	0.368
*cEVR*	13 (81.3%)	5 (62.5%)	4 (100.0%)	0.425
***SVR***	**13 (81.3%)**	**5 (62.5%)**	**4 (100.0%)**	**0.262**
**IL28B T/C-patients (n = 60) [n]**	**38**	**21**	**1**	
*RVR*	10 (26.3%)	4 (19.0%)	1 (100.0%)	0.180
*cEVR*	21 (55.3%)	9 (42.9%)	1 (100.0%)	0.339
***SVR***	**21 (55.3%)**	**10 (47.6%)**	**1 (100.0%)**	**0.651**
**IL28B T/T-patients (n = 13) [n]**	**6**	**7**	-	
RVR	1 (16.7%)	2 (28.6%)	-	0.612
cEVR	3 (50.0%)	4 (57.1%)	-	0.558
**SVR**	**3 (50.0%)**	**1 (14.3%)**	**-**	**0.221**

Virologic response rates (rapid virological response, complete early virological response and sustained virological response) to PEGIFN/RBV by PNPLA3-SNP and IL28B-SNP; reported as number (percentage) of patients; Abbreviations: PNPLA3 (patatin-like phospholipase domain-containing protein 3), IL28B (interleukin 28B), RVR (rapid virological response), cEVR (complete early virological response), SVR (sustained virological response).

101 (57.1%) patients received antiviral therapy with PEGIFN/RBV. Among these, fifty-eight (57.4%) patients achieved sustained virologic response (SVR).

When comparing the SVR rates between patients with major, minor or no PNPLA3 risk allele after grouping patients according to their IL28B-genotype, there was no statistically significant difference: Both RVR rates (p = 0.37 for IL28B-C/C, p = 0.12 for IL28B-T/C, and p = 0.62 for IL28B-T/T) and cEVR rates (p = 0.43 for IL28B-C/C; p = 0.34 for IL28B-T/C, and p = 0.56 for IL28B-T/T) were similar across different PNPLA3 genotypes. Moreover, SVR rates (p = 0.26 for IL28B-C/C, p = 0.65 for IL28B-T/C and p = 0.22 for IL28B-T/T) were similar in all PNPLA3 groups.

## Discussion

Our study provides novel data on the impact of a PNPLA3 risk allele on liver fibrosis progression in HIV/HCV coinfection. In addition, this is also the first study to investigate the influence of the PNPLA3 on development of hepatic steatosis and portal hypertension in a large cohort of the special population of HIV/HCV coinfected patients.

Stratification of patients according to their individual risk of developing cirrhosis and portal hypertension has become a major health issue in HCV monoinfection and HIV/HCV coinfection. In the era of IFN-free, DAA-based therapies that can cure up to 95% of all HCV infections [6–8], the main limitation of initiating HCV-treatment has shifted from side effects and contraindications [35] to socioeconomic issues (costs) [8]. Thus, predictors of disease progression to optimize treatment prioritization are of important clinical relevance.

Fibrosis progression rate has been extensively studied in HIV/HCV coinfected patients within the last years [4,9,24,36] but genetic predictors for high FPR have yet to be investigated [36]. Although paired liver biopsy samples are still considered a gold standard to assess fibrosis progression [36], non-invasive tests have been validated for staging liver fibrosis in HIV/HCV coinfection and have developed into the standard tool in clinical practice [37]. In our study, we used both liver biopsy and transient elastography to assess liver fibrosis [38].

PNPLA3 SNPs were associated with increased liver fibrosis progression in HCV-monoinfection [39,40], HIV-monoinfection [41] and other etiologies of liver disease [20]. Interestingly, even though we observed an increased inflammatory activity in patients harbouring a risk allele as reflected by the elevated transaminases, in our study there was no significant impact of a PNPLA3 risk allele on fibrosis progression or on the development of advanced fibrosis—neither in an univariable nor in a multivariable model. While PNPLA3 was not associated with advanced fibrosis in our cohort of HIV/HCV coinfected patients, HCV-GT3, age and BMI were independently associated with advanced liver fibrosis. Although there was only a trend toward a higher risk of advanced liver fibrosis in patients with low CD4+ nadir, we would not challenge the relevance of CD4+ nadir as a determinant of liver disease progression. As liver disease progression in this special population of HIV/HCV coinfected patients is accelerated due to several mechanisms including an altered cytokine environment, higher levels of reactive oxygen species [42], impaired cellular immune system [5], and other consequences of HIV infection [43], it seems that in HIV/HCV coinfection an additional “hit” of PNPLA3 high risk genotype does not have a significant impact. This hypothesis is supported by the similar prevalence of portal hypertension among the different PNPLA3 genotypes.

The presence of hepatic steatosis accelerates liver fibrosis progression in HCV monoinfection as well as in HIV/HCV coinfection [44,45]. Several risk factors for liver steatosis, such as increased body weight, HCV-GT3, insulin resistance and other hallmarks of the metabolic syndrome [46] have already been established. In our HIV/HCV coinfected patient cohort we used the CAP^™^-Module of the FibroScan^®^ device for evaluation of steatosis [47]. CAP has been validated for patients with HCV monoinfection by Sasso et al. showing that a CAP-value ≥222dB/m identifies patients with significant hepatic steatosis [30]. In accordance with previous studies describing a prevalence of hepatic steatosis of 24–75% in HIV/HCV coinfected patients [46,48,49], 52.9% of our patients had significant hepatic steatosis. When comparing patient groups with different PNPLA3-genotype, we could not find a difference in the prevalence of hepatic steatosis across the groups. A recent study by Ampuero et al. [33] showed a significant influence of PNPLA3 G-allele on steatosis in HCV monoinfected patients being infected with HCV GT1, but not in those with HCV GT3 infection. Following this finding we performed another analysis after exclusion of HIV/HCV GT3 patients—but the results were comparable to those obtained from the entire cohort. The majority of our patients was young, had non-adipose BMI, and only very few patients had diabetes mellitus (n = 10, 5.65%). We might speculate that in non-adipose young patients with HIV/HCV coinfection other causes of liver steatosis such as HCV GT3 or metabolic factors might play a more important role than the PNPLA3 genotype. However, the conclusion that the presence of a PNPLA3 risk allele has no impact on development of significant liver steatosis in HIV/HCV coinfection is limited by the low patient number in this subgroup analysis.

Even in the era of DAAs, the former standard therapy for chronic hepatitis C (CHC)-treatment, PEGIFN/RBV, remains important and will continue to be used in resource-limited settings. Well-established factors predicting virological response to PEGIFN/RBV-treatment in HIV/HCV coinfection include HCV GT, HCV-RNA, IL28B [26], liver fibrosis, insulin resistance, low-density lipoprotein (LDL)-cholesterol-levels[50], low CD4+ counts and nadirs [51] and y-glutamyltransferase (GGT) levels [50]. Previous studies showed no impact of PNPLA3 high risk alleles on treatment outcome in unselected CHC patients [23], but in a selected subgroup of HCV GT 1/4-patients with advanced fibrosis [52]. Thus, we also analyzed SVR rates in our HIV/HCV coinfected patients receiving PEGIFN/RBV therapy stratified by IL28B and PNPLA3-polymorphisms. No statistically significant differences in treatment outcomes were observed. However, all 4 patients with the PNPLA3 major risk genotype G/G in the group with IL28B C/C genotype achieved SVR, indicating that the presence of a PNPLA3 risk allele does at least not impair virological response in patients with favorable IL28B genotypes.

In summary, the PNPLA3 (rs738409) SNP is neither associated with faster liver fibrosis progression, nor with advanced fibrosis nor significant hepatic steatosis in HIV/HCV coinfection. Moreover, HIV/HCV coinfected patients with a PNPLA3 risk allele are not at increased risk for development of portal hypertension and show similar SVR rates to PEGIFN/RBV therapy.

As the prevalence of the PNPLA3-high risk genotype (G/G) is reported to be very low in European CHC collectives (3–12% [23,39,53], being 5.1% in our cohort and 5.4% in another Austrian collective[40]) and the presence of a high-risk PNPLA3 allele did not show any impact on liver disease progression in HIV/HCV coinfection, we would not recommend to use PNPLA3 genotyping in daily clinical routine.

## Abbreviations

HIV: human immunodeficiency virus
HCV: hepatitis C virus
SNP: single nucleotide polymorphism
PNPLA3: patatin-like phospholipase domain-containing protein 3
IL28B: interleukin 28B
CAP^™^: Controlled Attenuation Parameter
HVPG: hepatic venous pressure gradient
PEGIFN: pegylated interferon
RBV: ribavirin
ESLD: end-stage-liver-disease
HCC: hepatocellular carcinoma
DAA: direct-acting antiviral
NAFLD: non-alcoholic fatty liver disease
ULN: upper limit of normal
IVDU: intravenous drug-use
cART: combined anti-retroviral therapy
FPR: fibrosis progression rate
SVR: sustained virological response
RVR: rapid virological response
cEVR: complete early virological response
CHC: chronic hepatitis C
